# Outline of the History of Malignant or Asiatic Cholera in New Orleans, La.

**Published:** 1892-10

**Authors:** Joseph Jones

**Affiliations:** Professor of Chemistry and Clinical Medicine, Tulane University of Louisiana, New Orleans, La.


### OUTLINE OF THE HISTORY OF MALIGNANT OR ASIATIC CHOLERA IN NEW ORLEANS, LA.

BY JOSEPH JONES, M. D., LL. D.,

Professor of Chemistry and Clinical Medicine, Tulane University of Louisiana, New Orleans, La.

It would be foreign to our purpose to enter fully into the history of this foreign pestilence, which has at various times reached the shores of Louisiana from the cities of Europe, across the waters of the Atlantic ocean and the gulf of Mexico. Asiatic cholera has played no insignificant part in the grand carnival of disease and death. In 1832, Asiatic cholera, in conjunction with yellow fever, swelled the mortality of New Orleans to 8,090, in a population of 55,084, and marked this year as the most terrible in the annals of this city, the death rate reaching the enormous proportion of 147.10 per 1,000 inhabitants. In 1832 the inhabitants of New Orleans were more than decimated, for more than one-seventh of their number were destroyed, chiefly by Asiatic cholera and yellow fever, acting in addition to the usual endemic and epidemic diseases.

The Charity Hospital affords the following statistics of Asiatic cholera :

| YEAR. | CASES. | DEATHS. |
|---|---|---|
| 1848 | 662 | 396 |
| 1849 | 1,813 | 1,122 |
| 1850 | 724 | 530 |
| 1851 | 382 | 292 |
| 1852 | 485 | 358 |
| 1853 | 194 | 115 |
| 1854 | 478 | 352 |
| 1855 | 351 | 225 |
| 1856 | 32 | 11 |
| 1857 | 1 | 1 |
| Total | 5,122 | 3,402 |

During 18 years, 1842–1860, per cent. of deaths, 66.4.

During the sixteen years following the American civil war, New Orleans has been comparatively exempt from Asiatic cholera, as shown by the statistics of the Charity Hospital, thus :

| YEAR. | CASES. | DEATHS. |
|---|---|---|
| 1866 | 300 | 237 |
| 1867 | 94 | 70 |
| 1868 | 15 | 9 |
| 1873 | 34 | 27 |
| Total (1864–1880) | 443 | 343 |

Per cent. of deaths, 77.4.

There were received into the Charity Hospital during thirty-four years (1842–1880) 5,565 cases of Asiatic cholera, of which 3,745 terminated fatally, giving a rate of mortality of 67.3 per cent.

The first authentic records which we have of the appearance of Asiatic cholera in New Orleans relate to the year 1832, when it occasioned 4,340 deaths out of a total of 8,090 deaths from all causes. Yellow fever claimed only 400 deaths in this most pestilential year in the annals of this city. The disease lingered through 1833, when it claimed 1,000 victims out of a total of 4,976 deaths. In 1832 the population of New Orleans was

55,084, and of this number more than one-seventh perished, giving a mortality from all causes of 147.10 per thousand inhabitants, and from Asiatic cholera 78.78, and from yellow fever 7.20.

Cholera appeared again in 1848 and destroyed 1,646 inhabitants, and continued its ravages for several years, the deaths being: 1849, 3,176; 1850, 1,448; 1851, 430; 1852, 1,329; 1853, 585; 1855, 883; 1856, 43; 1857, 24; 1858, 26; 1859, 27; 1860, 30; 1861, 12; 1863, 4; 1864, 5; 1865, 9; total deaths from Asiatic cholera during a period of nineteen years (1844–1865), 9,678.

Cholera appeared again in 1866, and continued its effects for three years. The deaths were as follows: 1866, 1,294; 1867, 581; 1868, 129; 1869, 4; 1870, 3; 1871, 6; 1873, 142; 1874, 6; 1875, 4; total deaths during fifteen years (1866–1880), 2,169.

It is evident from the preceding statistics that, during a period of thirty-four years (1844–1880), Asiatic cholera destroyed 11,847 of the citizens of New Orleans; and if we add to this number the deaths occasioned by this disease in 1832 and 1833, we have a grand total of 17,187 deaths.

Those charged with the conduct of the sanitary affairs of this city and State should view those 17,187 dead citizens as victims to a foreign or exotic pestilence, imported from the shores of Europe in the cargoes and passengers of ships.

The State of Louisiana can protect herself and the entire valley only by maintaining, at all times and under all circumstances, a vigilant quarantine at the mouth of the Mississippi river; and all parties who, under the guise of promoting commerce, endeavor to destroy an efficient quarantine should be regarded as *public enemies*.

It is probable that the mortality occasioned by Asiatic cholera was even far in excess of these figures, for we find, upon careful examination of the mortuary records of New Orleans, that during a period of thirty-four years (1844–1880), the deaths from bowel affections were as follows: Cholera morbus, 889; cholera infantum, 2,408; teething, 3,430; gastritis, 743; enteritis,

6,915; dysentery, 7,097; diarrhœa, 8,289; total from these diseases, 29,771.

During the same period of thirty-four years, yellow fever occasioned 28,739 deaths.

It is evident, therefore, that the so-called ordinary bowel affections, diarrhœa, dysentery, cholera morbus, enteritis, gastritis and teething, actually caused a larger number of deaths in New Orleans than yellow fever, and if we add the 11,847 deaths caused by Asiatic cholera, we have a grand total of 41,618 deaths from those diseases in which derangements of the gastro-intestinal mucous membrane forms the most prominent symptom.

The continuous and fearful mortality from this class of diseases must be diminished by improved domestic and general sanitation. The great essentials for sanitary reform, for the diminution of the number and fatality of the cases of bowel affections in New Orleans must be based upon—

1. Thorough drainage of the entire parish of Orleans, and especially that portion occupied by the city of New Orleans.

2. The prompt removal of all fæcal matters out of the limits of the city.

3. The daily removal of garbage.

4. Systematic and thorough cleansing of private premises, public buildings, factories, markets, streets and gutters.

5. The filling up with sand and gravel all low lots.

6. The supply in abundance to all the citizens of New Orleans, at a small cost, of pure filtered river water.

7. The daily inspection of the markets, of milk, meats, fish, fruit and all food supplies.

156 *Washington Avenue, New Orleans.*

